# Alien chromosome segment from *Aegilops speltoides* and *Dasypyrum villosum* increases drought tolerance in wheat via profuse and deep root system

**DOI:** 10.1186/s12870-019-1833-8

**Published:** 2019-06-07

**Authors:** M. Djanaguiraman, P. V. V. Prasad, J. Kumari, S. K. Sehgal, B. Friebe, I. Djalovic, Y. Chen, K. H. M. Siddique, B. S. Gill

**Affiliations:** 1 Department of Agronomy, Kansas State University, Manhattan, Kansas, 66506 USA; 2 ICAR-National Bureau of Plant Genetic Resources, ICAR, New Delhi, 110 012 India; 3 Department of Agronomy, Horticulture and Plant Science, South Dakota State University, Brookings, SD 57007 USA; 4 Wheat Genetic Resource Center, Department of Plant Pathology, Kansas State University, Manhattan, Kansas, 66506 USA; 5 Institute of Field and Vegetable Crops, Novi Sad, Serbia; 6 The UWA Institute of Agriculture and School of Agriculture and Environment, The University of Western Australia, Perth, WA 6009 Australia; 7 Department of Crop Physiology, Tamil Nadu Agricultural University, Coimbatore, 641 003 India

**Keywords:** Alien substitution lines, Drought, Individual seed weight, Root angle, Root length, Seed set percentage, Wheat

## Abstract

**Background:**

Recurrent drought associated with climate change is a major constraint to wheat (*Triticum aestivum* L.) productivity. This study aimed to (i) quantify the effects of addition/substitution/translocation of chromosome segments from wild relatives of wheat on the root, physiological and yield traits of hexaploid wheat under drought, and (ii) understand the mechanism(s) associated with drought tolerance or susceptibility in wheat-alien chromosome lines.

**Methods:**

A set of 48 wheat-alien chromosome lines (addition/substitution/translocation lines) with Chinese Spring background were used. Seedling root traits were studied on solid agar medium. To understand the influence of drought on the root system of adult plants, these 48 lines were grown in 150-cm columns for 65 d under full irrigation or withholding water for 58 d. To quantify the effect of drought on physiological and yield traits, the 48 lines were grown in pots under full irrigation until anthesis; after that, half of the plants were drought stressed by withholding water for 16 d before recording physiological and yield-associated traits.

**Results:**

The alien chromosome lines exhibited altered root architecture and decreased photochemical efficiency and seed yield and its components under drought. The wheat-alien chromosome lines T5DS·5S#3L (TA5088) with a chromosome segment from *Aegilops speltoides* (5S) and T5DL^.^5 V#3S (TA5638) with a chromosome segment from *Dasypyrum villosum* (5 V) were identified as drought tolerant, and the drought tolerance mechanism was associated with a deep, thin and profuse root system.

**Conclusions:**

The two germplasm lines (TA5088 and TA5638) could be used in wheat breeding programs to improve drought tolerance in wheat and understand the underlying molecular genetic mechanisms of root architecture and drought tolerance.

**Electronic supplementary material:**

The online version of this article (10.1186/s12870-019-1833-8) contains supplementary material, which is available to authorized users.

## Background

Wheat (*Triticum aestivum* L.) is a major staple crop, and its production needs to increase by ~ 38% by 2050 to feed the growing population [1]. Among the environmental changes expected in the future, climate models predict an increase in the variability of precipitation leading to an increased frequency and intensity of drought across the globe [2]. Globally, ~ 79% of wheat harvesting regions show grain yield variability due to changes in precipitation and temperature [3]. Therefore, the enhancement of wheat drought tolerance (i.e., maintenance of high yields) is a key challenge in wheat improvement programs [4].

Bread wheat is an allohexaploid species (AABBDD genomes) that arose ∼8000 years ago [5], from spontaneous hybridization of the tetraploid wheat *T. turgidum* L. (AABB genomes) with the diploid goatgrass *Aegilops tauschii* Coss. (DD genomes) [6, 7]. Wheat has several primary, secondary and tertiary relatives spread across several genera including *Triticum*, *Aegilops (Ae.)*, *Dasypyrum, and Thinopyrum/Agropyron.* Wild relatives of wheat are native to semi-arid zones of West and Central Asia and are therefore well adapted to various abiotic stresses [8, 9]. Studies have shown that the introduction of alien chromosome segments from wild relatives into wheat have increased tolerance or resistance to drought [10], high temperatures [11], salinity [12], and water-logging [13]. Similarly, introduction of alien chromosome segments from wild relatives into wheat have improved pest resistance and yield [14, 15]. Waines and Ehdaie [15] and Yediay et al. [16] have sucessufully introgressed the genes associated with stem rust (*Puccinia graminis*) and powdery mildew (*Blumeria graminis* f. sp. *tritici*) from rye (*Secale cereale* L.) to wheat germplasm, which resulted in increased stem rust and powdery mildew tolerance. The wheat lines showing rye-wheat translocation (1RS) had a positive performance on yield, root morphology, and water and nitrogen use efficiency [10]. Sequencing, expression studies, functional annotations, and high-throughput genomics analysis can accelerate allele mining for several traits in sets of chromosome segment substitution lines. Though genetic diversity in wild wheat is a useful resource for trait discovery, only limited numbers of wild relatives have been exploited due to the weedy morphology and low fertility of interspecific hybrids [17]. To utilize the genes and alleles conferring abiotic and biotic stress tolerance from wild relatives of wheat, different genetic materials in the form of addition, substitution, and translocation lines have been developed after laborious efforts in the last few decades [18]. Some translocation lines of wheat-*Agropyron elongatum* and wheat-rye have been studied for their response to drought stress [4, 10, 19], and lines with rye translocation 1BL-1RS have been used in cultivars across the world to enhance drought tolerance.

Roots appear to be the most relevant organ for breeding drought tolerance, yet limited research is available on root traits due to the difficulty of phenotyping and measuring under both field and controlled environments. Root system architecture refers to the spatial and temporal configuration of roots in the soil. Understanding the variability and contribution of specific root traits can help in the development of drought*–*tolerant genotypes. In most crops, genetic variation for root traits has been reported using mapping populations or wild relatives [20, 21]. To our knowledge, genetic variation for root architecture in the alien chromosome addition/substitution or translocation lines involving *Aegilops* species and/or *D. villosum* under control and/or drought stress is not well understood. Placido et al. [4] reported that *Ag. elongatum* 7DL.7EL translocations in wheat improved seed yield under water limiting conditions by increasing root biomass. Similarly, a rye*–*wheat centric chromosome translocation 1RS.1BL increased yield under drought by enhancing root biomass [10, 19]. The yield advantage of the 1RS translocation line under water-limited conditions was partly associated with deceased root diameter, increased root length density and biomass [10]. Lukaszewski [22] observed three centric translocations, namely 1RS.1AL, 1RS.1BL, and 1RS.1DL, in Pavon 76 wheat background with greater root biomass and higher grain yield under irrigated and drought conditions. The genetic analyses of 1RS.1BS recombinant breakpoints in Pavon 76 indicated that the distal 15% of the physical length of chromosome 1RS might carry the gene(s) for better rooting ability and root morphological traits [19].

Relatively large root systems under drought can increase water uptake to alleviate the drought stress effect [23]. In contrast, since roots are a major sink for assimilates, reducing root biomass can increase the availability of assimilates for aboveground parts including grain [24]. Wheat production in India, Australia, and the United States represents a cross-section of global spring wheat production. In India, wheat is grown during winter (in the post-rainy season) and hence, is dependent on water stored in deep soil layers. Similarly, in north eastern Australia and the United States, spring wheat relies largely on stored soil moisture [25]. Hence, targeting access to deeper soil moisture with selected root traits in wheat is critical. Several root morphological traits like root angle, root diameter, and root length density have been associated with increased root system depth and water uptake [25–27]. Research into the physiological basis of drought tolerance in wheat is well established; however, the contribution of alien chromosome addition/substitution or translocation lines to drought tolerance is not fully understood. Osipova et al. [28] mapped quantitative trait locus (QTL) underlying chlorophyll fluorescence parameters and antioxidant enzyme on chromosome 7D of wheat under drought. Bobo et al. [29] reported that a spring wheat substitution line with the 3D chromosome from winter wheat had reduced quantum yield of photosystem II (ΦPSII) under low light intensities. The substitution lines of durum–Chinese Spring (1B with 1D and 3B with 3D chromosomes) had increased photosynthetic rates compared to the check, indicating that the D genome had a positive interaction with photosynthetic rate [30]. However, Haour-Lurton and Planchon [31] identified inhibitory effects of specific chromosomes of the D genome on photosynthesis. Wheat–barley addition lines (7H and 7HL) had higher ΦPSII, stomatal conductance and photosynthetic rates under salinity stress [32]. In another study, under terminal high temperature stress, the substitution lines of Chinese Spring (CS)–*D. villosum* (4 V.3 L, 6 V.3 L, and 5S.3L) had increased chlorophyll index, ΦPSII, individual seed weight and seed yield per plant than the check namely Chinese Spring [11]. Drought during gametogenesis causes a maximum reduction in grain number by inducing floret sterility in wheat and other crops [33]. However, drought during grain filling decreases individual grain weight [33, 34].

A combination of measuring and quantifying root phenotype and whole-plant physiological traits was used in this study to understand the drought-adaptive advantage introduced by the alien chromosome segment into wheat that can benefit wheat breeding programs focused on enhancing drought tolerance. The objectives of this research were to (i) quantify the effects of addition/substitution/translocation of chromosome segments from wheat wild relatives on the root, physiological and yield traits of hexaploid wheat under drought; and (ii) understand the mechanism(s) associated with drought tolerance or susceptibility in wheat-alien chromosome lines.

## Results

### Principal component analyses

**Fig. 1:**
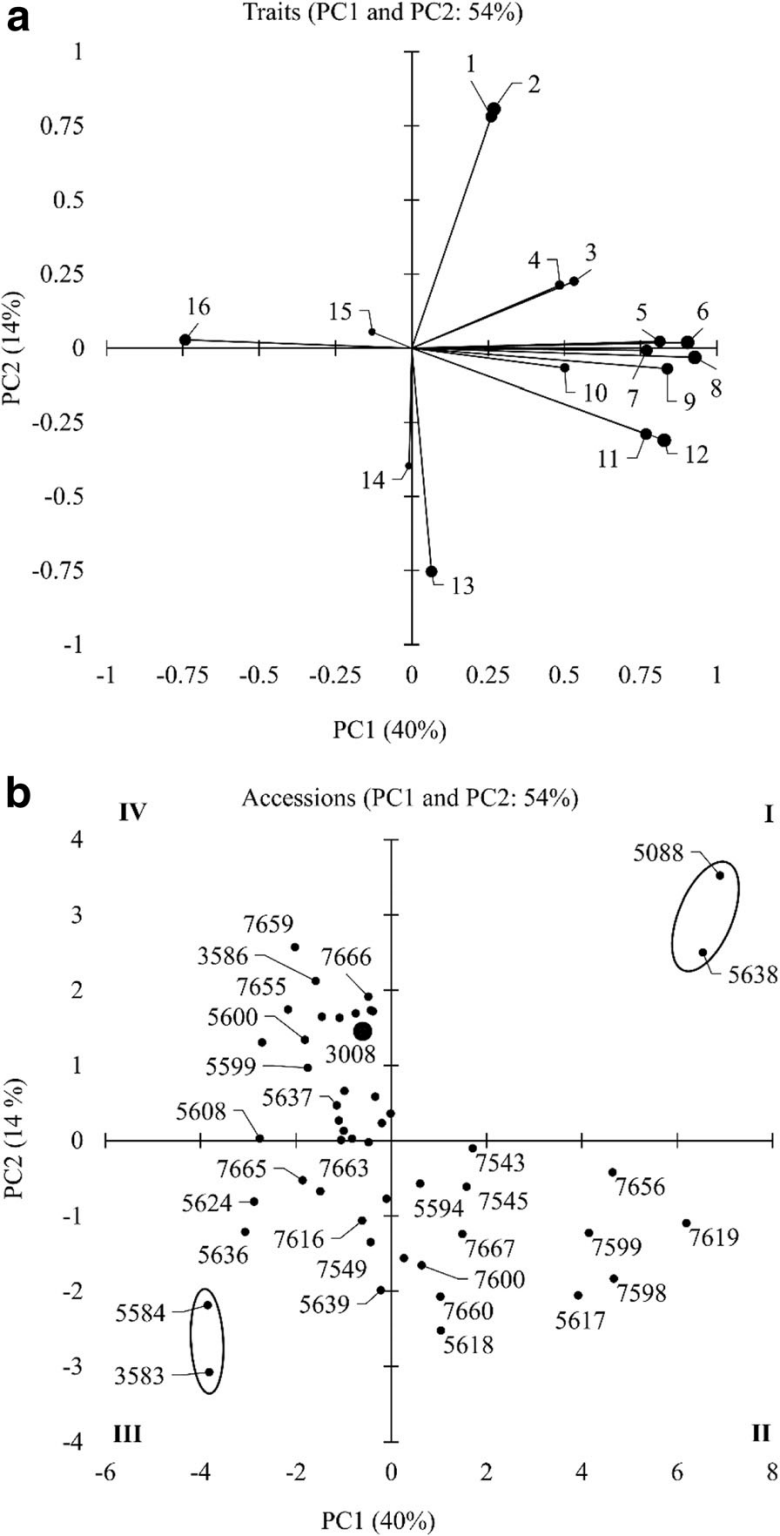
Principal component analysis of chromosome substitution lines for identification of traits governing drought tolerance and genotypes with drought tolerance. (**a**) The principal component scores (PC1 and PC2) for identification of traits governing drought tolerance; (**b**) classification of 48 wheat-alien chromosome lines and the CS check based on factor scores of PC1 and PC2 for 16 trait variables. Description for (**a**) factor loading values for variables are indicated by black arrows radiating from the centre showing the direction (angle) and magnitude (length) and the contribution of the variables shown as the size of the circle, (**b**) four distinct groups are formed among the lines and the CS check (white circle). Legends for (**a**) 1: maximum root length (cm); 2; maximum root length: shoot length ratio (unitless); 3: number of seeds per plant, 4; number of seminal roots; 5: seed set percentage, 6: total root length (cm); 7: quantum yield of PSII (ΦPSII; unitless); 8: seed yield (g plant^−1^); 9: harvest index (%); 10: chlorophyll index (SPAD units);11: root volume (cm^3^); 12: individual seed weight (mg); 13: seminal root angle (°); 14: root diameter (mm); 15: seedling root length (cm),; and 16: thylakoid membrane damage (F_o_/F_m_ ratio; unitless). (**b**) I, II, III and IV indicates four groups. 3008, Chinese Spring

The first two principal components (PC) from the principal component analysis (PCA) represented 54% of the variability, with PC1 explaining 40% of the variance. For PC1, the major contributors to the variance were seed yield per plant (13%) and total root length (12%). For PC2, the major contributors were maximum root length:shoot length ratio (28%) and maximum root length (26%) (Fig. 1a). The 48 wheat-alien chromosome lines and the CS check were grouped into four (I to IV) groups based on the PCA (Fig. 1b). The lines in group I (TA5088 and TA5638) and II (e.g., TA7619 and TA7598) were classified as tolerant and moderately tolerant, respectively, to drought (Fig. 1b). The lines in group III (e.g., TA3583 and TA5584) and IV (CS and TA7659) were classified as susceptible and moderately susceptible, respectively to drought. Among the lines, the highest variation along PC1 was caused by TA5088 (7%) followed by TA5638 (6.5%), and in PC2 the highest variation was caused by TA5088 (3.5%) followed by TA7659 (2.5%) (Fig. 1b). The representative tolerant (TA5088 and TA5638) and susceptible genotypes (TA3583 and TA5584) for different traits were compared with a background check (CS) for root, physiological and yield traits to understand the mechanism of tolerance or susceptibility.

### Experiment 1. Genetic variability in seedling root traits

The size of a plant’s root system is a key trait that can affect the uptake of water from the soil. The 48 lines had significant (*P* > 0.001) genetic variability for the number of seminal roots (2.6–6.3), seedling root length (6.8–23.7 cm) and root angle (38.6–59.3°); their corresponding mean values were 4.2, 18.1 cm and 48.7° (Table 1). Among the accessions, TA5088 and TA5638 had more seminal roots (6.3 vs. 2.6) and a lower root angle (av. 39 vs. 46°) than the CS check (Table 2). Accessions TA3583 and TA5584 had longer seedling roots (av. 22 vs. 10.9 cm) and a greater root angle (59 vs. 46°) than the CS check but no significant difference in the number of seminal roots (Table 2).

**Table 1:** Range, mean and least significant difference (LSD) for the number of seminal roots, seedling root length (cm), and root angle (°) among the wheat–-alien chromosome lines (Experiment 1)

Trait	Range	Mean	LSD
Number of seminal roots	2.6–6.3	4.2	1.1***
Seedling root length (cm)	6.8–23.7	18.1	2.5***
Root angle (°)	38.6–59.3	48.7	6.3***

*** indicates *P* ≤ 0.001

**Table 2:** Mean values for the number of seminal roots, seedling root length (cm) and root angle (°) of the six highest (top) and six lowest (bottom) ranked wheat-alien chromosome lines along with the CS check (Experiment 1)

Rank	Number of seminal roots	Seedling root length (cm)	Root angle (°)
Top 6			
	TA5088 (6.3 ± 0.52)	TA5636 (23.0 ± 0.55)	TA7660 (59.3 ± 1.20)
	TA5638 (6.3 ± 0.51)	TA5584 (22.6 ± 0.79)	TA3583 (59.0 ± 0.33)
	TA7667 (6.3 ± 0.67)	TA7655 (22.0 ± 0.10)	TA5584 (59.0 ± 1.53)
	TA7599 (6.0 ± 0.11)	TA7598 (22.0 ± 0.29)	TA7598 (57.0 ± 3.48)
	TA7619 (5.0 ± 0.58)	TA3583 (21.6 ± 0.26)	TA5594 (56.0 ± 2.31)
	TA7688 (5.0 ± 0.58)	TA6656 (21.5 ± 0.88)	TA5618 (55.0 ± 1.53)
Bottom 6			
	TA3583 (3.3 ± 0.33)	TA7660 (13.4 ± 0.14)	TA7657 (41.3 ± 0.33)
	TA5584 (3.3 ± 0.33)	TA7616 (12.1 ± 0.27)	TA7544 (41.0 ± 2.41)
	TA5637 (3.3 ± 0.33)	TA5616 (12.0 ± 0.13)	TA5624 (40.7 ± 0.58)
	TA7543 (3.3 ± 0.33)	TA7620 (11.7 ± 0.11)	TA5638 (40.0 ± 2.67)
	TA7547 (3.3 ± 0.33)	TA7667 (10.1 ± 0.50)	TA6656 (38.7 ± 1.86)
	TA5657 (2.6 ± 0.33)	TA7549 (6.8 ± 0.11)	TA5088 (38.3 ± 2.66)
Check (CS)			
	3008 (2.6 ± 0.33)	3008 (10.9 ± 0.58)	3008 (46.0 ± 0.58)
LSD	1.1	2.5	6.3

Genotypes were ranked based on numerical values. Values in parentheses are means ± standard error of the respective trait. *CS* Chinese Spring, *LSD* least significant difference

### Experiment 2. Genetic variability in shoot and root traits under drought

There were significant (*P* > 0.001) effects of genotype, drought, and their interaction for all growth and root-related traits; except the genotype × drought interactions for plant height and shoot dry weight per plant. Under drought, maximum root length ranged from 58.5–164.5 cm, total root length ranged from 1826 to 10,873 cm, maximum root length:shoot length ratio ranged from 0.95–2.90 and root volume ranged from 0.55–4.60 cm^3^ in the 48 substitution lines (Table 3). Averaged across the lines, drought significantly (*P* > 0.001) reduced plant height (7%), maximum root length (26%), maximum root length:shoot length ratio (21%), total root length (7%), root diameter (12%), root volume (23%), number of tillers per plant (33%), and stem dry weight per plant (42%), relative to the control indicating that both shoot and root growth are affected by drought stress (Table 3).

**Table 3:** Range, mean and least significant difference (LSD) for plant height (cm), maximum root length (rooting depth; cm), maximum root length:shoot length ratio (unitless), total root length (cm), root diameter (mm), root volume (cm^3^), tiller number (plant^− 1^) and stem dry weight (g plant^− 1^) of wheat–alien chromosome lines under irrigation (control) and drought stress (water withheld for 58 d) (Experiment 2)

Trait	Irrigated condition		Drought stress		LSD for stress
	Range	Mean	Range	Mean	
Plant height (cm)	44.0–102.5	63.4	39.6–97.0	58.7	2.8***
Maximum root length (rooting depth; cm)	108.0–186.0	146.9	58.5–164.5	108.7	5.8***
Maximum root length:shoot length ratio (unitless)	1.15–3.20	2.39	0.95–2.90	1.90	0.11***
Total root length (cm)	1559–11,599	5131	1826–10,873	4747	276***
Root diameter (mm)	0.19–0.32	0.26	0.17–0.29	0.23	0.009***
Root volume (cm^3^)	0.70–5.50	2.6	0.55–4.60	2.0	0.2***
Tiller number (plant^−1^)	3.5–12.5	5.8	1.5–12.0	3.9	0.45***
Stem dry weight (g plant^− 1^)	0.41–6.40	2.6	0.28–4.70	1.5	0.25***

*** indicates *P* ≤ 0.001

Accessions TA5088 and TA5638 had significantly (*P* > 0.001) higher maximum root length (av. 161 vs. 124 cm), maximum root length:shoot length ratio (av. 2.8 vs. 2.2) and total root length (av. 10,517 vs. 3768 cm) than the CS check under drought (Table 4). However, the lines TA3583 and TA5584 had lower maximum root length (av. 73 vs. 124 cm), maximum root length:root length ratio (av. 1.2 vs. 2.2) and total root length (av. 2459 vs. 3768 cm) than the CS check under drought (Table 4). Compared to CS, higher maximum root length and total root length was observed in the accessions TA5088 and TA5638 due to its narrow root angle.

**Table 4:** Mean values for maximum root length (rooting depth; cm), maximum root length:shoot length ratio (unitless) and total root length (cm) of the six highest (top) and six lowest (bottom) ranked wheat–alien chromosome lines along with the CS check under irrigation (control) and drought stress (water withheld for 58 d) (Experiment 2)

Rank	Maximum root length (cm)			Maximum root length:shoot length ratio (unitless)			Total root length (cm)		
	Line	Irrigated	Drought	Line	Irrigated	Drought	Line	Irrigated	Drought
Top 6									
	TA7659	155.0 ± 3.2	164.5 ± 4.2	TA5088	3.10 ± 0.05	2.90 ± 0.11	TA5638	7643 ± 487	10,873 ± 44
	TA5088	171.5 ± 6.5	161.5 ± 4.2	TA5638	2.35 ± 0.04	2.70 ± 0.06	TA5088	11,600 ± 2329	10,162 ± 51
	TA5638	152.0 ± 7.0	161.0 ± 7.0	TA3586	3.15 ± 0.05	2.65 ± 0.41	TA7598	4325 ± 804	8643 ± 1312
	TA7662	116.0 ± 4.0	145.0 ± 14.1	TA7594	2.60 ± 0.04	2.55 ± 0.42	TA7656	7235 ± 184	8511 ± 590
	TA7657	114.5 ± 14.5	135.0 ± 7.8	TA7655	2.10 ± 0.05	2.55 ± 0.11	TA7619	3544 ± 66	8423 ± 86
	TA7663	109.5 ± 11.5	133.0 ± 14.3	TA3585	3.15 ± 0.35	2.50 ± 0.14	TA7545	6042 ± 566	7840 ± 68
Bottom 6									
	TA5584	174.5 ± 2.4	75.5 ± 2.9	TA5584	2.75 ± 0.19	1.40 ± 0.20	TA3586	3124 ± 1608	2500 ± 308
	TA5618	168.0 ± 2.0	75.5 ± 10.3	TA7663	1.25 ± 0.11	1.35 ± 0.35	TA5584	3634 ± 530	2491 ± 135
	TA7600	137.0 ± 2.0	75.5 ± 8.3	TA5636	2.41 ± 0.19	1.30 ± 0.20	TA3583	5619 ± 568	2427 ± 89
	TA7620	149.5 ± 23.8	74.0 ± 16.7	TA3583	2.60 ± 0.29	1.10 ± 0.40	TA5636	2873 ± 1408	2378 ± 39
	TA3583	159.0 ± 23.8	71.0 ± 5.7	TA7620	2.25 ± 0.37	1.05 ± 0.15	TA7616	4385 ± 1324	2198 ± 105
	TA5639	152.50 ± 2.4	58.5 ± 20.1	TA5639	2.21 ± 0.11	0.95 ± 0.45	TA5599	6147 ± 412	1826 ± 258
Check (CS)									
	TA3008	151.0 ± 24.5	124.0 ± 4.9	TA3008	2.36 ± 0.15	2.20 ± 0.16	TA3008	4195 ± 336	3768 ± 822
LSD (Genotype)			28.8			0.55			1368
LSD (Genotype x Stress)			5.8			0.11			276

Genotypes were ranked based on the numerical values under drought stress. Values are means ± standard error of the respective trait. *CS* Chinese Spring, *LSD* least significant difference

### Experiment 3. Genetic variability in physiological and yield traits under drought

There were significant (*P* > 0.001) effects of genotype, drought, and their interaction for chlorophyll index (SPAD units), thylakoid membrane damage (F_o_/F_m_ ratio; unitless), ΦPSII (unitless), electron transport rate (μmol electrons m^− 2^ s^− 1^), seed set percentage, seed yield (g spike^− 1^), seed number plant^− 1^, individual seed weight (mg seed^− 1^), seed yield (g plant^− 1^) and harvest index (%) (Table 5). There was wide genetic variability for various physiological and yield traits under control and drought conditions. Among the 48 lines, thylakoid membrane damage and the ΦPSII ranged from 0.255–0.425, and 0.105–0.465, respectively, under drought. Similarly, seed set percentage and number of seeds per plant ranged between 6.7–59.8% and 2.0–89.0 plant^− 1^, respectively. Individual seed weights ranged from 3.9–59.9 mg with a mean of 22.8 mg under drought. Seed yield and harvest index ranged between 0.02–2.0 g plant^− 1^ and from 0.18–29.5%, respectively, under drought. Irrespective of the line, drought significantly (*P* > 0.001) reduced chlorophyll index (26%), ΦPSII (43%), electron transport rate (44%), seed set percentage (52%), seed yield per spike (63%), number of seeds (60%), individual seed weight (23%), seed yield per plant (66%), and harvest index (54%) but increased thylakoid membrane damage (67%) compared to the control. Overall, the result indicates drought stress had negative effect on both photosynthetic efficiency and yield associated traits. Comparing both photosynthetic efficiency and yield traits the later was found to be more sensitive than former.

**Table 5:** Range, mean and least significant difference (LSD) for chlorophyll index (SPAD units), thylakoid membrane damage (F_o_/F_m_ ratio; unitless), quantum yield of PSII (ΦPSII; unitless), electron transport rate (μmol electrons m^−2^ s^− 1^), seed set percentage, seed yield (g spike^− 1^), seed number (plant^− 1^), individual seed weight (mg seed^− 1^), seed yield (g plant^−1^) and harvest index (%) of wheat–alien chromosome lines under irrigation (control) and drought stress (water withheld for 16 d) (Experiment 3). *** indicates *P* ≤ 0.001

Trait	Irrigated condition		Drought stress		LSD for stress
	Range	Mean	Range	Mean	
Chlorophyll index (SPAD units)	30.0–54.2	41.8	19.9–49.3	31.1	0.38***
Thylakoid membrane damage (F_o_/F_m_ ratio; unitless)	0.140–0.260	0.203	0.255–0.425	0.340	0.003***
Quantum yield of PSII (ΦPSII; unitless)	0.345–0.590	0.462	0.105–0.465	0.262	0.005***
Electron transport rate (μmol electrons m^−2^ s^−1^)	197.3–297.6	242.2	50.6–215.3	135.8	3.0***
Seed set percentage	44.5–84.0	68.0	6.7–59.8	32.9	0.84***
Seed yield (g spike^−1^)	0.12–1.91	0.92	0.02–1.26	0.34	0.02***
Seed number (plant^−1^)	23.5–133.2	57.1	2.0–89.0	23.0	2.1***
Individual seed weight (mg seed^− 1^)	6.0–56.0	29.6	3.9–59.9	22.8	1.3***
Seed yield (g plant^− 1^)	0.17–3.5	1.6	0.02–2.0	0.54	0.04***
Harvest index (%)	2.5–31.3	15.2	0.18–29.5	7.0	0.44***

*** indicates *P* ≤ 0.001

The ranking of wheat-alien chromosome lines based on the numerical values of different physiological traits are in Table 6. The data indicate that drought stress increases damage to the thylakoid membrane, and reduces chlorophyll content and ΦPSII in both the deep and shallow rooting accessions, however, the damage was lower in the deep rooting accessions than in the shallow rooting accessions. The lines TA5088 and TA5638 had a higher chlorophyll index and ΦPSII and lower thylakoid membrane damage than the CS check under drought (Table 6), and higher seed set percentages (55.8 and 59.8%, respectively), individual seed weights (44.5 and 45.5 mg seed^− 1^, respectively) and seed yields (2.27 and 1.61 g plant^− 1^, respectively) than the CS check (34.0%, 13.2 mg seed^− 1^ and 0.51 g plant^− 1^, respectively); both lines were grouped with the six best-performing genotypes under drought (Table 7). The lines TA3583 and TA5584 had lower seed set percentages (15.9 and 15.7%, respectively), individual seed weights (9.4 and 7.8 mg seed^− 1^, respectively), and seed yields (0.07 and 0.07 g plant^− 1^, respectively) than the CS check under drought. The higher seed set percentage and individual seed weight was observed in the accessions TA5088 and TA5638 compared to CS, resulting in higher seed yields.

**Table 6:** Mean values for chlorophyll index (SPAD units), thylakoid membrane damage (F_o_/F_m_ ratio; unitless) and quantum yield of PSII (ΦPSII; unitless) of six highest (top) and six lowest (bottom) ranked wheat–alien chromosome lines along with CS check under irrigation (control) and drought stress (water withheld for 16 d) (Experiment 3)

Rank	Chlorophyll index (SPAD units)			Thylakoid membrane damage (F_o_/F_m_ ratio; unitless)			Quantum yield of PSII (ΦPSII; unitless)		
	Line	Irrigated	Drought	Line	Irrigated	Drought	Line	Irrigated	Drought
Top 6									
	TA7598	54.25 ± 0.65	49.30 ± 0.80	TA5608	0.200 ± 0.012	0.425 ± 0.012	TA7656	0.58 ± 0.02	0.47 ± 0.03
	TA7600	40.30 ± 1.20	43.15 ± 1.45	TA7659	0.195 ± 0.011	0.420 ± 0.021	TA7619	0.53 ± 0.01	0.45 ± 0.02
	TA7543	42.10 ± 0.50	38.25 ± 0.15	TA5599	0.215 ± 0.011	0.415 ± 0.011	TA7543	0.49 ± 0.01	0.43 ± 0.02
	TA7655	42.30 ± 0.40	38.15 ± 0.45	TA5600	0.185 ± 0.012	0.405 ± 0.012	TA5088	0.59 ± 0.01	0.40 ± 0.01
	TA5638	47.15 ± 1.65	38.10 ± 0.55	TA5584	0.195 ± 0.013	0.405 ± 0.011	TA5638	0.51 ± 0.01	0.39 ± 0.01
	TA5088	46.15 ± 0.35	36.90 ± 1.30	TA3583	0.225 ± 0.011	0.400 ± 0.011	TA7545	0.47 ± 0.01	0.37 ± 0.01
Bottom 6									
	TA5637	43.60 ± 1.30	23.80 ± 0.45	TA7599	0.190 ± 0.010	0.260 ± 0.012	TA7544	0.53 ± 0.01	0.13 ± 0.01
	TA3583	47.85 ± 1.76	21.45 ± 1.15	TA5088	0.195 ± 0.012	0.255 ± 0.016	TA7667	0.37 ± 0.01	0.13 ± 0.01
	TA7657	34.85 ± 0.75	20.85 ± 0.75	TA5617	0.220 ± 0.015	0.255 ± 0.013	TA6656	0.43 ± 0.01	0.12 ± 0.01
	TA5640	30.00 ± 0.45	20.75 ± 0.35	TA5638	0.155 ± 0.016	0.255 ± 0.012	TA3583	0.46 ± 0.01	0.11 ± 0.01
	TA7664	34.50 ± 1.10	19.95 ± 0.36	TA7619	0.210 ± 0.012	0.255 ± 0.012	TA5584	0.52 ± 0.01	0.11 ± 0.01
	TA5584	39.35 ± 0.45	19.60 ± 1.02	TA7656	0.195 ± 0.015	0.255 ± 0.011	TA5636	0.47 ± 0.01	0.11 ± 0.01
Check (CS)									
	TA3008	32.3 ± 1.10	21.05 ± 0.45	TA3008	0.220 ± 0.011	0.355 ± 0.011	TA3008	0.47 ± 0.03	0.32 ± 0.01
LSD (Genotype)			1.89			0.017			0.020
LSD (Genotype x Stress)			1.86			0.003			0.005

Genotypes were ranked based on numerical values under drought stress. Values are means ± standard error of the respective trait. *CS* Chinese Spring, *LSD* least significant difference

**Table 7:** Mean values for seed set percentage, individual seed weight (mg seed^−1^) and seed yield (g plant^− 1^) of six highest (top) and six lowest (bottom) ranked wheat–alien chromosome lines along with CS check under irrigation (control) and drought stress (water withheld for 16 d) (Experiment 3)

Rank	Seed set percentage			Individual seed weight (mg seed^−1^)			Seed yield (g plant^− 1^)		
	Line	Irrigated	Drought	Line	Irrigated	Drought	Line	Irrigated	Drought
Top 6									
	TA5638	82.4 ± 1.9	59.8 ± 2.1	TA7598	37.1 ± 1.8	60.0 ± 4.5	TA5088	3.42 ± 0.13	2.27 ± 0.07
	TA7545	69.1 ± 2.9	55.8 ± 1.3	TA7619	43.6 ± 2.2	56.9 ± 3.7	TA7619	2.49 ± 0.14	1.78 ± 0.08
	TA5088	68.5 ± 1.5	55.8 ± 2.4	TA7599	56.0 ± 3.4	55.0 ± 3.6	TA5638	2.19 ± 0.10	1.61 ± 0.11
	TA7619	66.4 ± 1.1	53.2 ± 1.1	TA5617	42.7 ± 3.8	50.4 ± 6.5	TA7598	1.88 ± 0.13	1.31 ± 0.07
	TA5617	66.5 ± 2.1	51.7 ± 3.9	TA5638	22.9 ± 1.0	45.5 ± 2.4	TA5617	1.63 ± 0.30	1.29 ± 0.10
	TA7656	63.8 ± 1.2	48.4 ± 1.6	TA5088	45.0 ± 1.6	44.5 ± 4.4	TA7599	1.69 ± 0.10	1.26 ± 0.07
Bottom 6									
	TA3583	72.3 ± 3.7	15.9 ± 1.5	TA3583	10.6 ± 0.4	9.4 ± 1.1	TA7665	0.76 ± 0.06	0.11 ± 0.01
	TA5584	66.0 ± 1.8	15.7 ± 1.0	TA7666	17.4 ± 2.3	9.2 ± 2.2	TA5584	0.29 ± 0.01	0.07 ± 0.01
	TA7544	61.4 ± 1.4	14.9 ± 1.2	TA7655	11.8 ± 2.2	8.8 ± 1.3	TA3583	0.262 ± 0.01	0.07 ± 0.01
	TA5624	71.2 ± 2.7	13.4 ± 1.1	TA5624	12.9 ± 0.7	8.6 ± 0.6	TA5624	0.45 ± 0.02	0.06 ± 0.02
	TA7688	68.0 ± 1.3	12.8 ± 1.1	TA5584	6.0 ± 0.4	7.8 ± 0.8	TA7544	0.72 ± 0.01	0.04 ± 0.01
	TA7655	48.2 ± 2.5	6.7 ± 1.2	TA7544	26.8 ± 1.0	6.7 ± 1.7	TA7655	0.30 ± 0.05	0.02 ± 0.01
Check (CS)									
	TA3008	68.53 ± 4.0	34.00 ± 2.6	TA3008	21.65 ± 1.2	13.2 ± 1.8	TA3008	2.46 ± 0.14	0.51 ± 0.04
LSD (Genotype)			4.10			6.5			0.21
LSD (Genotype x Stress)			0.84			1.3			0.04

Genotypes were ranked based on the numerical values under drought stress. Values are means ± standard error of the respective trait. *CS* Chinese Spring, *LSD* least significant difference. The top and bottom six genotypes are wheat–alien chromosome lines

### Relationship among root, physiological and yield traits

**Fig. 2:**
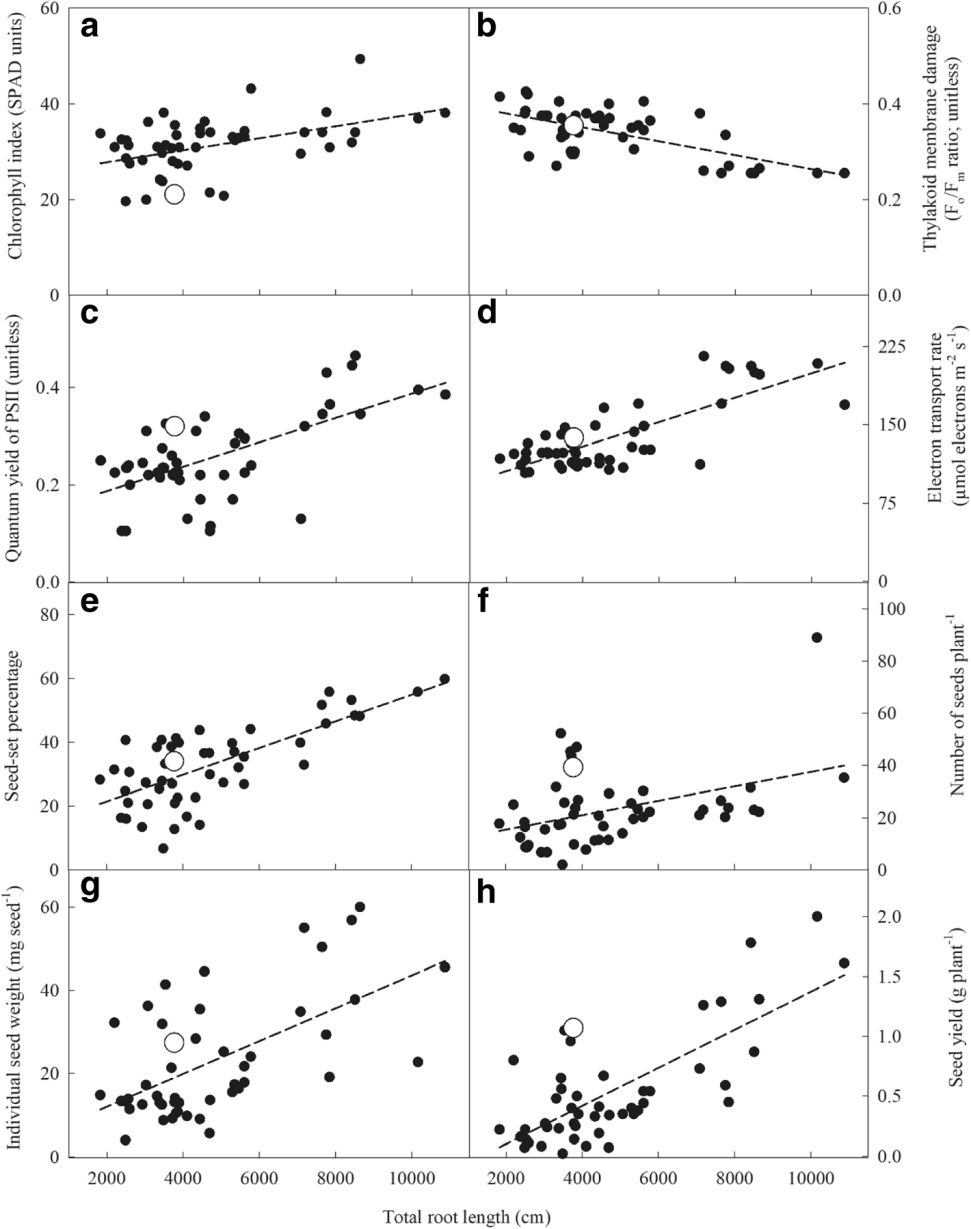
Chlorophyll index (SPAD units), thylakoid membrane damage (F_o_/F_m_ ratio; unitless), quantum yield of PSII (ΦPSII; unitless), electron transport rate (μmol electrons m^−2^ s^− 1^), and components of seed yield per plant as a function of total root length (cm) under drought stress in wheat-alien chromosome lines (water withheld for 16 d). The CS check is shown as a white circle. The curve is fitted by a linear function. (**a**) chlorophyll index, y = 25.3 + 0.0013 x; *r*^2^ = 0.21 (*P* < 0.01); (**b**) thylakoid membrane damage, y = 0.409–0.000014x; *r*^2^ = 0.39 (*P* < 0.01); (**c**) quantum yield of PSII, y = 0.137 + 0.000024x; *r*^2^ = 0.39 (*P* < 0.01); (**d**) electron transport rate, y = 82.4 + 0.011x; *r*^2^ = 0.60 (*P* < 0.01); (**e**) seed set percentage, y = 13.083 + 0.0042x; *r*^2^ = 0.52 (*P* < 0.001); (**f**) seed number per plant, y = 9.959 + 0.0028x; *r*^2^ = 0.16 (*P* < 0.01); (**g**) individual seed weight, y = 4.142 + 0.0039x; *r*^2^ = 0.35 (*P <* 0.001) and (**h**) seed yield per plant, y = − 0.217 + 0.0002x; *r*^2^ = 0.54 (*P* < 0.001)

Total root length was positively associated with chlorophyll index (*r*^2^ = 0.21; Fig. 2a), ΦPSII (*r*^2^ = 0.39; Fig. 2c), electron transport rate (*r*^2^ = 0.60; Fig. 2d), seed set percentage (*r*^2^ = 0.52; Fig. 2e), number of seeds per plant (*r*^2^ = 0.16; Fig. 2f), individual seed weight (*r*^2^ = 0.35; Fig. 2g) and seed yield per plant (*r*^2^ = 0.54; Fig. 2h) in the wheat–alien chromosome lines under drought. However, thylakoid membrane damage had a negative association with total root length (*r*^2^ = 0.39; Fig. 2b). The lines with more total root length had higher seed set percentage, individual seed weights and seed yields per plant under drought (Fig. 2e, g, h). Although all the above-mentioned traits were associated with total root length, the association between total root length and electron transport rate and seed yield per plant was higher than other traits indicating that these traits might be physiologically related.

**Fig. 3:**
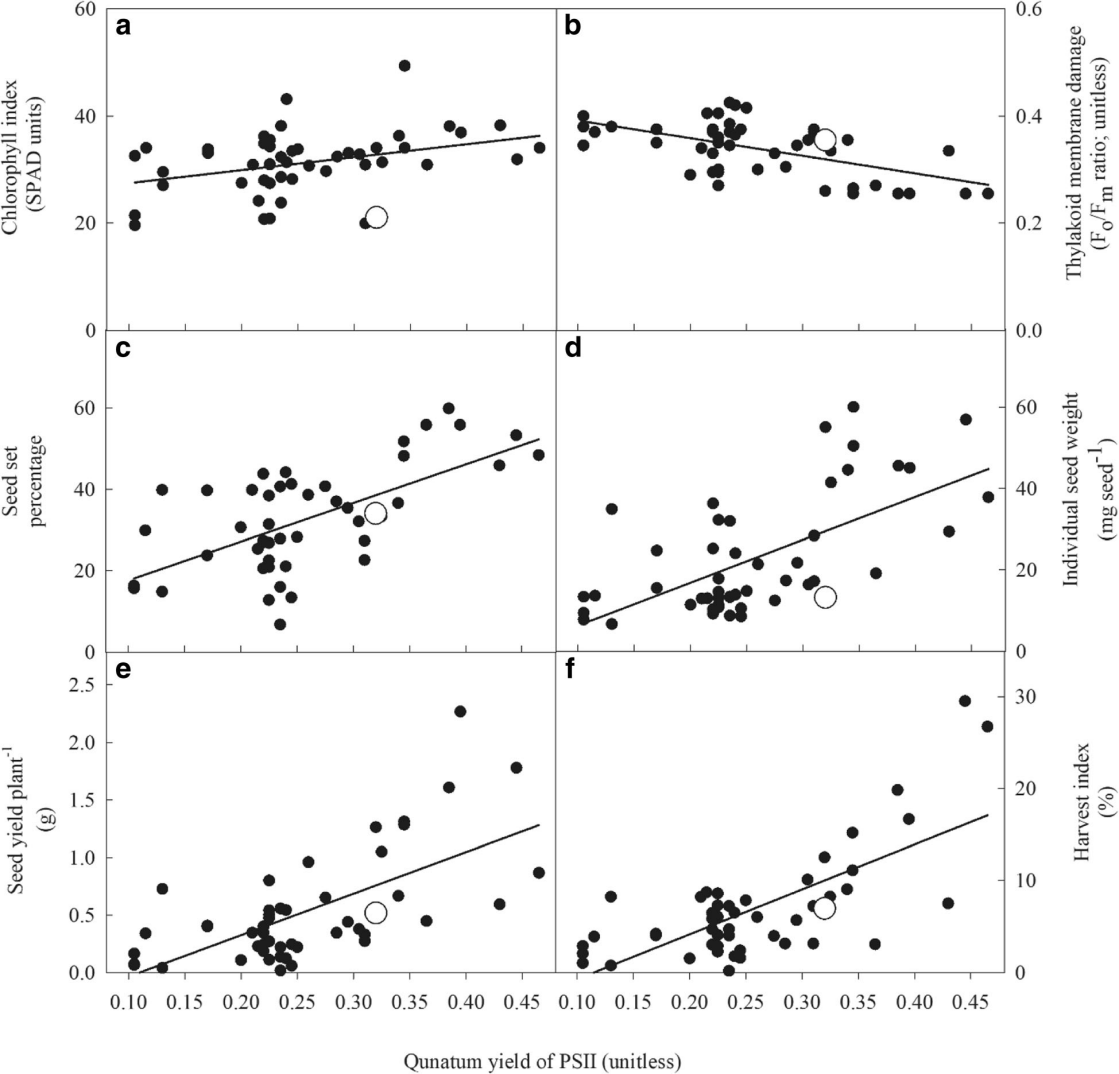
Chlorophyll index (SPAD units), thylakoid membrane damage (F_o_/F_m_ ratio; unitless) and components of seed yield per plant as a function of quantum yield of PSII (ΦPSII; unitless) under drought stress in wheat-alien chromosome lines (water withheld for 16 d). The CS check is shown as a white circle. The curve is fitted by a linear function. (**a**) chlorophyll index, y = 25.0 + 24.1x; *r*^2^ = 0.13 (*P* < 0.05); (**b**) thylakoid membrane damage, y = 0.424–0.328x; *r*^2^ = 0.33 (*P* < 0.001); (**c**) seed set percentage, y = 8.1 + 94.7x; *r*^2^ = 0.42 (*P* < 0.001); (**d**) individual seed weight, y = − 4.21 + 105.2x; *r*^2^ = 0.39 (*P <* 0.001); (**e**) seed yield per plant, y = − 0.396 + 3.61x; *r*^2^ = 0.42 (*P* < 0.001) and (**f**) harvest index, y = − 5.61 + 48.83x; *r*^2^ = 0.49 (*P* < 0.001)

The ΦPSII had a positive relationship [co-efficient of determination (*r*^2^) ≥ 0.39] with seed set percentage, individual seed weight, seed yield per plant and harvest index (Fig. 3c-f) but a negative relationship with thylakoid membrane damage (*r*^2^ = 0.33; Fig. 3b) under drought in the wheat-alien chromosome lines. The lines with a higher ΦPSII had higher seed set percentages, individual seed weights and seed yields per plant (Fig. 3c-e).

## Discussion

This study demonstrated that (i) alien segments in the wheat background have altered root architecture and largely reduced photochemical efficiency and seed yield components under drought, (ii) accessions TA5088 and TA5638 with alien chromosome segments from *Ae. speltoides* (5S) and *D. villosum* (5 V) in CS wheat, respectively were drought tolerant, and (iii) the drought tolerance mechanism is associated with a deep, thin and profuse root system.

Root traits play a significant role in plants exposed to drought [35]. Plant capacity to extract soil moisture is a key factor determining drought adaptation, which is likely to result from the improved exploration of soil water [36]. PCA identified accessions TA5088 and TA5638 as drought tolerant (Fig. 1b) and accessions TA3583 and TA5584 as drought susceptible (Fig. 1b) based on 16 root, shoot and yield traits. Accession TA5088 has the long arm of chromosome 5S from *Ae. speltoides* and TA5638 have the short arm of chromosome 5 V from *D. villosum*. *Ae. speltoides* and *D. villosum* are wild relatives of wheat and rich sources of genetic variation for resistance to drought [37, 38]. The effect of 5S and 5 V translocation in bread wheat was evident from the rooting depth, total root length, and root diameter. In general, cereal roots do not have a vascular cambium and must develop more root length to generate new xylem tissues for water transport [39]. Hence, in water-limiting situations, the genotypes with increased root length had more opportunity for water uptake. This has been confirmed in rice, wheat and maize as deep rooting with profuse branching and small root diameters are associated with drought tolerance [25–27, 40]. In contrast, accession TA3583 with a monosomic addition for chromosome 4S from *Ae. searsii* and TA5584 with the chromosome 7 J arm translocated from *Th. Intermedium* had wider root angles with sparse root branching at depth and increased root diameter, which is associated with topsoil foraging that eventually results in drought susceptibility [25]. Hence, it is postulated that the gene(s) associated with deep rooting and profuse branching are present in 5S of *Ae. speltoides* and 5 V of *D. villosum* and the gene(s) associated with shallow rooting and sparse branching are localized at 4S and 7 J of *Ae. searsii* and *Th. intermedium,* respectively.

Studying root architecture and associating it with crop performance under drought can help to identify proxy traits for improving drought tolerance in wheat [36, 40]. The present study clearly showed that total root length and ΦPSII could be used as a proxy trait to evaluate drought tolerance in wheat because of its strong, positive relationship between seed set percentage and seed yield per plant under drought (Fig. 3a). Under drought, the chlorophyll index, ΦPSII and electron transport rate decreased in all 48 lines but the thylakoid membrane damage increased (Table 5). The structural and functional alterations in chloroplast under drought namely damages to thylakoid membranes may lead to chlorophyll loss because chlorophyll is primarily located in the thylakoid membranes [41]. The decreased ΦPSII and electron transport rate under drought shows the occurrence of photoinhibition [42, 43]; and it could be associated with the generation of excess excitation energy [43]. The observed genetic variability for chlorophyll index and ΦPSII in the wheat-alien chromosome lines falls within the range reported by Pour-Aboughadareh et al. [44]. Under drought stress, the ΦPSII in TA5088 and TA5638 increased more (av. 25%) than CS check. Chromosome substitution lines developed from synthetic wheat in the CS background showed that genes regulating F_o_, F_m_ and ΦPSII values might be located on the 3A, 4D and 7A chromosomes [45]. Higher chlorophyll content and lower membrane damage under drought stress in the tolerant genotype of wheat have been reported [46]. Overall, the study suggests that photoinhibition of PSII under drought lead to reduced photochemical efficiency in the wheat-alien chromosome lines and the CS check.

Drought stress during flowering significantly reduced seed set percentage and individual seed weight in all wheat-alien chromosome lines (Table 7), which is attributed to the loss of gametic function and decreased seed filling rate and duration, respectively [47]. Significant genotypic differences were observed for seed set percentage and individual seed weight, which reflects the ability of the alien chromosome segment to endure drought. The wheat-alien chromosome lines TA5088 and TA5638 had higher seed set percentage and individual seed weight than the CS check. This is in accordance with the finding of Fang et al. [48] that drought-tolerant wheat genotypes had more seeds per plant and higher individual seed weights than drought-susceptible genotypes.

In wheat, the root biomass in subsoil correlates positively with individual seed weight and grain yield under drought stress [48]. Similarly, the ΦPSII correlated positively with grain yield under drought stress [49]. The robust association of these variables with grain yield ratifies that yield is a function of water content and the photosynthetic process. The alien chromosome segments (5S of *Ae. speltoides* and 5 V of *D. villosum*) altered the root system and increased PSII photochemistry, which increased grain yield under drought stress. Two wheat-alien chromosome lines (TA5088 and TA5638) will be valuable germplasm for breeding for drought tolerance because of its deep root system and high reproductive success. Breeders can mobilize these translocated segments into adapted local germplasm and estimate the genetic value of these traits in their environment. It is possible that the same alien fragment introgressed into different wheat genotypes could exert different effects on resistance to drought. Therefore, the genetic background and interaction of these genetic factors need to be further investigated. Further, genetic and molecular studies need to be conducted to unravel the genetic factors controlling root- and shoot- related traits in the long arm of chromosome 5S of *Ae. speltoides* and the short arm of chromosome 5 V of *D. villosum*, and their contribution to drought tolerance in wheat.

## Conclusions

Alien chromosome segments altered root architecture and decreased photochemical efficiency, seed set percentage, individual seed weight, and seed yield per plant in 48 lines of wheat under drought. The wheat-alien chromosome lines TA5088 and TA5638, having chromosome or chromosome segment from *Ae. speltoides* (5S) and *D. villosum* (5 V), respectively, were identified as drought tolerant. These two lines had deep, thin and profuse root system under drought stress, which can help to alleviate drought stress by enhancing access to water. Total root length and the ΦPSII were associated with higher seed set percentage and seed yield per plant under drought stress; hence, these traits can be used as proxy traits for improving drought tolerance in wheat. TA5088 and TA5638 lines will be valuable germplasm for understanding the molecular mechanism(s) and breeding of wheat for improved drought tolerance.

## Methods

Seeds of 48 CS wheat-alien chromosome lines (chromosome addition/substitution /translocation) belonging to *Ae. speltoides* (4)*, Ae. searsii* (3), *Ae. longissima* (5)*, Ae. peregrina* (8)*, Ae. geniculata* (14)*, Th. intermedium* (3), *L. racemosus* (1) and *D. villosum* (10) and the background CS (check) were obtained from the Wheat Genetic Resources Center, Kansas State University, Manhattan, Kansas. The wheat–alien chromosome lines are derivatives of CS (*T. aestivum*)–*Ae. speltoides, Ae. searsii*, *Ae. longissima, Ae. peregrina, Ae. geniculata, Th. Intermedium, L. racemosus* and *D. villosum*. Details of the lines used in this study are in Additional file 1: Table S1.

### Experiment 1. Genetic variability in seedling root traits

Twenty seeds of each of the 48 lines and the check were surface-sterilized using 10% (v/v) sodium hypochlorite for 5 min and then washed with deionized water for three times. The seeds were germinated in Petri plates using filter paper (Whatman no 42) moistened with five mL of deionized water for two days. Square Petri plates (12 × 12 × 1.7 cm, L × W × H) was used in this study. A slit was made using a scissor on the sides of Petri plates top and bottom and covered with cellophane tape (Staples® Invisible Tape, 2 × 3200 cm, Staples, Manhattan, KS). Sterilized agar (Sigma Type A; 2% w/v) was poured into Petri plates. The Petri plates were sealed with cellophane tape. On the third day, uniformly sized seedlings (radicle emerged) were selected and placed one per Petri plates containing agar in the slit with the radicle facing downward. The Petri plates were incubated at 25 ± 1 °C for 5 d [50]. After the stipulated time, the root angle of individual root axes of seminal roots, counting upwards from the primary seminal root (or radicle), was measured at 3 cm from the seed relative to a vertical line passing through the stem base [50]. Seedling root length was estimated using the cigar roll method [51].

### Experiment 2. Genetic variability in the shoot and root traits under drought

The experiment was conducted in the greenhouse facilities at the Department of Agronomy, Kansas State University, Manhattan, KS to evaluate the variability of root system characteristics among 48 CS wheat–alien chromosome lines and a CS check. Before starting the experiment, the greenhouse was fumigated for one hour using an automated sulfur vaporizer (Rosemania, Franklin, TN) to avoid powdery mildew attack. The rooting medium was Turface MVP® (PROFILE Products LLC, Buffalo Grove, IL), which had a bulk density of 577 ± 32 kg m^− 3^. Turface is a non-swelling illite and silica clay that allows easy separation of roots. Plants were grown in polyvinyl chloride (PVC) columns with an inside diameter of 7.5 cm and height of 150 cm. The bottom of the PVC columns had plastic caps with a central hole of 0.5 cm diameter for drainage. Before sowing, each PVC column was filled with Turface and fertilized with 4 g of Osmocote (a slow-release fertilizer with 19:6:12 gravimetric percentages of N:P_2_O_5_:K_2_O; Scotts, Marysville, OH, USA) and 1 g of Marathon 1% G (granular; a.i.: Imidacloprid:1-[(6-chloro-3-pyridinyl)methyl]-N-nitro-2-imidazolidinimine; OHP, Inc., Mainland, PA, USA) which were evenly mixed with the Turface in the top 2 cm. Three seeds of a single genotype were sown at 4 cm depth in each PVC column. After emergence, the columns were thinned to one plant per column. Plants were maintained at 24/14 °C (daytime maximum/nighttime minimum temperature) from sowing to harvest (65 d after sowing) at a photoperiod of 16 h (natural light and supplemental fluorescent lights). The fungicide, Bumper 41.8 EC (emulsifiable concentrate; a.i.: Propiconazole:1-[[2-(2,4 dichlorophenyl)-4-propyl-1,3-dioxolan-2-yl]methyl]-1H-1,2,4–triazole; 1.2 mL L^− 1^; Makhteshim Agan of North America, Inc., Raleigh, NC, USA) was applied on 20 d after sowing to prevent powdery mildew attack. The control plants were maintained at 100% field capacity from sowing to final harvest with drip irrigation. For the drought treatment, plants were stressed by withholding water from day 7 to final harvest (65 days). The duration of drought stress was 58 days at which the genotypes were at the booting stage (Feekes’ stage 10). Our earlier experiments on wheat have indicated that plants grown in Turface have shown the drought stress effects (leaf rolling symptoms) after 45 days of stress imposition [52]. In another experiment withholding water for 41 days in common bean grown in Turface have decreased chlorophyll index by 5% and increased thylakoid membrane damage by 13% [53]. Therefore, in the present study water was withheld for 58 days (first drought symptoms of leaf rolling were observed about 45 d after withholding water) to cause a significant effect on biomass and root growth.

Plant height and number of tillers per plant were measured one day before harvest. Plant height was determined as the distance from the Turface level to the ligule of the youngest leaf and expressed in cm. At harvest, the PVC columns were gently inverted at about 140° to allow the contents (Turface and plants with entire root system) of the column to slip out. The shoots were cut at the base; and the aboveground biomass was oven dried at 65 °C to constant weight, weighed, and expressed as g plant^− 1^. The roots were carefully separated from the Turface without breaking the root system. The roots were laid on a flat surface and straightened to measure the maximum root length (rooting depth; from the base of the stem to the tip of the root system) and expressed in cm. The root system was carefully washed in water to remove any adhering Turface, placed between the moist paper towels, sealed in Ziploc bags (S.C. Johnson & Sons, Inc. Racine, WI, USA), transported to the laboratory, and stored at 4 °C.

The root system of each plant was sliced into 30-cm-long portions; each portion was submerged in water in a tray (20 × 15 × 2 cm; L × W × H), carefully spread to minimize root overlap, and scanned using an Epson photo scanner (Epson Perfection V700 with 600 dpi resolution, Epson, Long Beach, CA, USA). Images of scanned roots were analyzed using the WinRHIZO Pro image system (Regent Instruments, Inc., Quebec City, QC, Canada) to estimate total root length, root diameter and root volume as explained by McPhee [54] and Singh et al. [55]. Total root length, root diameter, and root volume were expressed in cm, mm and cm^3^, respectively. The shoots were oven-dried at 60 °C for 7 d to determine shoot dry weight. Maximum root length:shoot length ratio for each genotype was calculated as the ratio of maximum root length to plant height [56].

### Experiment 3. Genetic variability in physiological and yield traits under drought

Experiment 3 was conducted to evaluate the variability in seed yield and its associated component traits among 48 CS wheat-alien chromosome lines and a CS check under drought conditions. Three seeds of a single genotype were sown in 1.6-L plastic pots [14 cm (height) × 50 cm (top perimeter) × 36 cm (bottom perimeter)] containing a 4:1 mixture of soil [well-drained Kennebec silt loam (a fine-silty, mixed, superactive, mesic Cumulic Hapludoll)] and sand, and 4 g of Osmocote Plus (N:P_2_O_5_:K_2_O = 15:9:12; Scotts, Marysville, OH, USA). Two large indoor growth chambers (Conviron Model CMP 3244, Winnipeg, Manitoba, Canada) were used for this experiment, each being 136 cm wide, 246 cm long, and 180 cm high. Twenty-five lines were randomly placed within each growth chamber (24 CS wheat–alien chromosome lines and a CS check), with four pots per line. The growth chambers were maintained at 24/14 °C (daytime maximum/nighttime minimum temperature) from sowing to physiological maturity at a photoperiod of 16 h and 70% relative humidity (RH), conditions that were optimal for the growth and development of the chromosome substitution lines [11]. The chambers were set at 70% RH to avoid drought due to rapid evapotranspiration. Air temperature and RH were monitored at 20-min intervals in the growth chambers throughout the experiment. Photosynthetically active radiation (PAR) of 680 μmol m^− 2^ s^− 1^ was provided by cool white fluorescent lamps (Philips Lighting Co., Somerset, NJ, USA). The PAR was monitored once a month with a Field Scout Light Sensor (Spectrum Technologies, Inc., Plainfield, IL, USA). Fourteen days after seedling emergence, plants were thinned and staked, leaving two plants per pot. Granular Marathon 1% (a.i.: Imidacloprid, 1-((6Chloro-3-pyridinyl) methyl)-N-nitro-2-imidazolidinimine) pesticide was applied to avoid infestation by sucking insect pests. Within a chamber, pots were randomly moved every 7 d to avoid any positional effects. The pots were kept in trays containing ~ 2 cm of water to avoid drought; i.e., the plants were maintained at 100% pot capacity. At the booting stage, the main stem of each plant in the pots was tagged for recording yield and associated traits. Miracle-Gro, a water-soluble fertilizer (N:P_2_O_5_:K_2_O = 24:8:16; Scotts Miracle-Gro Products, Inc., Marysville, OH, USA) was added to the irrigation water (according to manufacturer’s instructions) once in every 7 d until anthesis (Feekes’ 10.5.1 stage). At anthesis, two pots of each line within each growth chamber were randomly assigned to the drought treatment. The drought stress was imposed by withholding water for 16 d. The other two pots were continuously irrigated and served as the control. At the end of the stress, the plants were re-watered and kept well-watered until physiological maturity.

Various physiological traits viz.*,* chlorophyll index, thylakoid membrane damage, ΦPSII, and electron transport rate were measured on tagged plants on 12 d after the drought treatment. Chlorophyll index was measured with a self-calibrating chlorophyll meter (SPAD-502, Spectrum Technologies, Plainfield, IL, USA) on the fully expanded flag leaf of the tagged main stem and expressed in SPAD units. Each time, data were taken in triplicate from the middle portion of the leaf, and the readings were averaged. Chlorophyll *a* fluorescence parameters were measured using a modulated fluorometer (OS-30p, Opti-Science Inc., Hudson, NH, USA). The minimal fluorescence (F_o_) and maximum fluorescence (F_m_) was measured in 30-min dark-adapted tagged flag leaves. Thylakoid membrane damage was determined as the ratio of F_o_/F_m_ (unitless). For other fluorescence measurements, the flag leaves were dark adapted for 2 h; the leaves were then continuously irradiated with white actinic light to measure the initial fluorescence in leaves acclimated to irradiation (F_o_’), steady-state fluorescence yield (F_s_), and maximum fluorescence yield (F_ms_) of irradiated leaves. Using the above parameters, the ΦPSII (ΦPSII = [F_ms_ – F_s_] / F_ms_; unitless) and apparent rate of photochemical transport of electrons through PSII (ETR = ΦPSII × PAR × 0.5 × 0.84; μmol electrons m^− 2^ s^− 1^) were calculated using the instrument software [57, 58], where ETR is electron transport rate, PAR is incident photosynthetically active radiation on a leaf, 0.5 corresponds to the proportion of absorbed quanta used by PSII reaction centers, and 0.84 represents the proportion of incident irradiance absorbed by the leaf [59].

The spike from the main tiller was tagged at the heading stage. At physiological maturity, the tagged and remaining spikes were harvested separately and dried in an incubator at 40 °C until constant weight. The vegetative biomass per plant was the weight of the oven dried (65 °C for 10 d) plant material without spikes and roots and expressed in gram. Individual spikelets were checked for grain by pressing the floret between the thumb and the index finger. Seed set percentage was estimated as the ratio of spikelets with grain to the total number of spikelets. The tagged spikes were hand threshed after drying, and the number of filled and unfilled grains were estimated for each spike. The remaining spikes were hand threshed, and the grains from these spikes counted and weighed to determine the number of grains per plant, grain weight per spike (g) and grain weight per plant (g). Individual grain weight was calculated by dividing grain weight per plant by number of grains per plant and expressed as mg seed^− 1^. Harvest index (%) was estimated as the ratio of grain yield to aboveground biomass.

### Data analyses

Statistical analyses were performed with SAS 9.4 [60]. Experiment 1 had a completely randomized design with four replications, and Experiments 2 and 3 had a split-plot design in randomized complete block design with two replications. The main plots were water regimes and sub-plots were accessions. Experiments 1 and 3 were repeated. Data from experiment 1 and 3 and their corresponding repeats were statistically analysed independently and found that there were no significant differences. Therefore, the data from both the experiments were pooled together for combined statistical analyses and the mean responses are presented. The PROC GLM procedure of SAS was used for data analysis. Standard errors are shown as an estimate of variability, and the means of various variables are separated for significance by Fisher’s least significant difference (LSD) at 5% significance level. The REG procedure in SAS was used to regress total root length and ΦPSII against other traits. Principal component analysis (PCA) based on the correlation matrix, was performed using XLSTAT-Pro software (AddinSoft, Inc., NY, USA) to identify influential traits under drought stress [61]. PCA biplots were plotted for the drought stress conditions using XLSTAT-Pro software to show the relationships among studied genotypes based on recorded traits.

## Additional file

Additional file 1: **Table S1.** List of wheat-alien chromosome lines and cultivars used in the present study [62–70]. (DOCX 17 kb)

## Abbreviations

CS: Chinese Spring
ETR: Electron transport rate
F_m_: Maximum fluorescence
F_ms_: Maximum fluorescence yield
F_o_: Minimal fluorescence
F_s_: Steady-state fluorescence yield
LSD: Least significant difference
PAR: Photosynthetically active radiation
PC: Principal components
PCA: Principal component analysis
PSII: Photosystem II
PVC: Polyvinyl chloride
QTL: Quantitative trait locus
RH: Relative humidity
SPAD: Soil Plant Analysis Development
ΦPSII: Quantum yield of photosystem II
