# Supplementary material for: Alien chromosome segment from Aegilops speltoides and Dasypyrum villosum increases drought tolerance in wheat via profuse and deep root system
Source: BMC Plant Biol. 2019 Jun 7;19:242. doi: 10.1186/s12870-019-1833-8 (PMC6554880; doi:10.1186/s12870-019-1833-8)
Supplement: Supplementary file 1 — Table S1. List of wheat-alien chromosome lines and cultivars used in the present study [62–70]. (DOCX 17 kb) [file 12870_2019_1833_MOESM1_ESM.docx]

Additional file 1: **Table S1.** List of wheat-alien chromosome lines and cultivars used in the present study.

| **Sl. No.** | **Accession^#^** | **Genetic information** | **Reference** |
| --- | --- | --- | --- |
| 1 | TA6653 | CS-AESPE DS 3S#3 [3A] | [62] |
| 2 | TA6654 | CS-AESPE DS 4S#3 [4B] | [62] |
| 3 | TA6656 | CS-AESPE DS 6S#3 [6B] | [62] |
| 4 | TA5088 | KS10WGGRC52: CS-AESPE DS [T5DS·5S#3L] | [9, 62] |
| 5 | TA3583 | CS-AESEA MA 4S^s^#1 | [63] |
| 6 | TA3585 | CS-AESEA DA 6S^s^#1 | [63] |
| 7 | TA3586 | CS-AESEA DA 7S^s^#1 | [63] |
| 8 | TA7543 | CS-AELON DA 1S^l^#3 | [64] |
| 9 | TA7544 | CS-AELON DA 2S^l^#3 | [64] |
| 10 | TA7545 | CS-AELON DA 3S^l^#3 | [64] |
| 11 | TA7547 | CS-AELON DA 5S^l^#3 | [64] |
| 12 | TA7549 | CS-AELON MA 7S^l^#3 | [64] |
| 13 | TA7594 | CS-AEPER DA 1S^v^#1 | [65] |
| 14 | TA7598 | CS-AEPER DA 5S^v^#1 | [65] |
| 15 | TA7599 | CS-AEPER DtA 6S^v^#1S | [65] |
| 16 | TA7600 | CS-AEPER DA 7S^v^#1 | [65] |
| 17 | TA7616 | CS-AEPER MA 3U^v^#1 | [65] |
| 18 | TA7617 | CS-AEPER DA 4U^v^#1 | [65] |
| 19 | TA7619 | CS-AEPER DA 6U^v^#1 | [65] |
| 20 | TA7620 | CS-AEPER DA 7U^v^#1 | [65] |
| 21 | TA7655 | CS-AEGEN DA 1M^g^#1 | [18] |
| 22 | TA7656 | CS-AEGEN DA 2M^g^#1 | [18] |
| 23 | TA7657 | CS-AEGEN DA 3M^g^#1 | [18] |
| 24 | TA7659 | CS-AEGEN DA 5M^g^#1 | [18] |
| 25 | TA7660 | CS-AEGEN DA 6M^g^#1 | [18] |
| 26 | TA7662 | CS-AEGEN DA 1U^g^#1 | [18] |
| 27 | TA7663 | CS-AEGEN DA 2U^g^#1 | [18] |
| 28 | TA7664 | CS-AEGEN DA 4U^g^#1 | [18] |
| 29 | TA7665 | CS-AEGEN DA 5U^g^#1 | [18] |
| 30 | TA7666 | CS-AEGEN MA 6U^g^#1 | [18] |
| 31 | TA7667 | CS-AEGEN MA 7U^g^#1 MtA 7U^g^#1L | [18] |
| 32 | TA7688 | CS-AEGEN DA 3U^g^#1 | [18] |
| 33 | TA5599 | WL711-AEGEN T5M^g^#2S·5M^g^#2L-5DL | [66] |
| 34 | TA5600 | WL711-AEGEN T5M^g^#2S·5M^g^#2L-5DL | [66] |
| 35 | TA5624L1 | KS12WGGRC59: CS-THIN DS T7BS·7S#3L [7B] | [67] |
| 36 | TA5584 | CS-THIN DS T7DS-7J#1L·7J#1S [7D] | [68] |
| 37 | TA5657 | KS13WGRC60VIL27-THIN T7DL·7J#1S T1DL^.^1V#3S[7D] | [68] |

Additional file 1: **Table S1**

Continued…

| **Sl. No.** | **Accession^#^** | **Genetic information** | **Reference** |
| --- | --- | --- | --- |
| 38 | TA5616 | CS-DVIL DS T1DL^.^1V#3S [1D] | [67] |
| 39 | TA5618 | CS-DVIL DS T6AL^.^6V#3S [6A] | [67] |
| 40 | TA5634 | CS-DVIL DS T2BS^.^2V#3L [2B] | [67] |
| 41 | TA5636 | CS-DVIL DS T3DL^.^3V#3S [3D] | [67] |
| 42 | TA5637 | CS-DVIL DS T3DS^.^3V#3L [3D] | [67] |
| 43 | TA5638 | CS-DVIL DS T5DL^.^5V#3S [5D] | [67] |
| 44 | TA5639 | CS-DVIL DS T7DL^.^7V#3S [7D] | [67] |
| 45 | TA5640 | CS-DVIL DS T7DS^.^7V#3L [7D] | [67] |
| 46 | TA5594 | CS-DVIL DS T4DS^.^4V#3L [4D] | [67] |
| 47 | TA5617 | KS12WGGRC57CS DVIL DS [T6AS·6V#3L] | [69] |
| 48 | TA5608L1 | CS-LRAC DS T7AL^.^7Lr#1S [7A] | [70] |
| 49 | TA3008 | Chinese Spring |  |

**^#^**Wheat Genetics Resource Center collection accession number of genetic stocks. Abbreviations: DA = disomic addition, DS = disomic substitution, MA = monosomic addition, MtA = monosomic telosome addition, T = translocation, CS = Chinese Spring, WL711 = Indian bread wheat cultivar, VIL27 = French wheat cultivar Vilmorin 27, AESPE = *Aegilops speltoides*, AESEA = *Aegilops searsiii*, AELON = *Aegilops longissima*, AEPER = *Aegilops perigrina*, AEGEN = *Aegilops geniculata*, THIN = *Thinopyrum intermedium*, DVIL = *Dasypyrum villosum,* LRAC = *Leymus racemosous*.
